# Study and personal resources of university students’ academic resilience and the relationship with positive psychological outcomes

**DOI:** 10.3389/fpsyg.2025.1517359

**Published:** 2025-02-05

**Authors:** Dalia Bagdžiūnienė, Irena Žukauskaitė, Laima Bulotaitė, Rūta Sargautytė

**Affiliations:** Institute of Psychology, Vilnius University, Vilnius, Lithuania

**Keywords:** university students, academic resilience, study resources, self-efficacy, engagement, well-being

## Abstract

**Introduction:**

Embarking on university-level studies is a period of new challenges for young people as they meet new academic demands, environments, and teaching systems. Academic resilience, defined as students’ capacity to endure challenges while sustaining optimism, positive thinking, and emotional stability, is crucial in enabling individuals to navigate academic difficulties and foster future success. In this context, developing the role of contextual and personal factors in university students’ academic resilience and its role in predicting positive psychological outcomes is crucial. This study, grounded in the Study Demands–Resources framework, sought to examine study-related characteristics and self-efficacy as resources that support students’ academic resilience. Additionally, it aimed to explore the connections between academic resilience and positive psychological outcomes, such as student engagement and well-being.

**Method:**

The convenience study sample included 350 students from Lithuanian universities: 79.14% were female, the mean age was 23.8 years (SD = 5.7). Students were in varying bachelor’s and master’s study programs. Data were collected using a self-administered online survey. Descriptive statistics, correlation, regression analyses, and structural equation modelling were applied for data analysis.

**Results and conclusion:**

The study found that students’ academic resilience was positively influenced by the characteristics of their study environment and self-efficacy. Furthermore, academic resilience was positively associated with student engagement and well-being. Our findings highlight the role of academic resilience in mediating the interplay between study-related resources, student engagement, and well-being. This research study features practical implications for enhancing university students’ academic resilience, engagement, and well-being by strengthening both study-related and personal resources.

## 1 Introduction

The modern labor market places high demands on graduates of higher education institutions as employers are looking for professionals who not only know their field well but are also motivated, ready for continuous improvement, able to quickly adapt to an unfamiliar environment, meet work requirements, and overcome professional challenges (Asefer and Abidin, 2021). Psychological resilience enables graduates to adapt to employers’ expectations at the beginning of their professional careers (Tomlinson, 2017) and to remain professionally active as they age and become more experienced (Berthung et al., 2021).

For many young people, tertiary education represents the final phase of formal education before transitioning into the labor market. This period can be particularly challenging for some, as they navigate the threshold of this life stage, adjust to a new academic environment and educational systems, and effectively handle the demands of their studies (Richardson et al., 2012). This underscores the importance of researching university students’ academic resilience, defined as their ability to navigate difficulties and challenges in achieving academic goals. Recent literature emphasizes that studies in this field have received increasing yet insufficient attention (Borazon and Chuang, 2023; Brewer et al., 2019).

Our study posits that personal resilience manifests through specific activities and is context-dependent. As circumstances evolve, the factors that activate resilience, the conditions that enhance it, and its impact on behavior or other psychological outcomes may also change. Teaching and learning conditions vary across educational institutions, and the significance of different contextual and personal characteristics for students’ academic resilience may differ accordingly.

While most studies on academic resilience have focused on primary and secondary school students (Gabrielli et al., 2022), their findings cannot be directly applied to the university context. This underscores the importance of analyzing the academic resilience of university students, along with its resources and effects, as they relate specifically to the university environment and study processes (Fullerton et al., 2021).

The importance of academic resilience is supported by research into its effects. The spectrum of positive outcomes at the individual level is broad, ranging from psychological adaptation to satisfaction with studies and indicators of academic progress. Research shows that resilience supports academic achievements (Pinkney and Walker, 2020), career decision-making and success (Shin and Kelly, 2015), and is also linked to student commitment (Martin and Marsh, 2006), satisfaction with studies (Meneghel et al., 2019), and psychological well-being (Cilliers and Flotman, 2016; Chen, 2016). Resilience may play a crucial role in helping students remain highly motivated during challenging circumstances, proactively manage the learning process, maintain a high level of engagement in their studies, and balance their academic responsibilities with other life domains. In this study, we analyzed the role of university students’ academic resilience in relation to two key psychological outcomes: engagement in studies and well-being.

In the context of academic resilience, it is important to consider cultural aspects, as reflected in studies conducted on university students across various countries. Sun and Liu (2023), using a sample of undergraduate, graduate, and postgraduate students from Henan Polytechnic University in China, demonstrated that academic resilience significantly influences academic engagement and educational attainment. Similarly, Guillen et al. (2022) conducted research across three higher education institutions in different Spanish regions and found that students’ resilience is strongly associated with academic engagement and performance. Bittmann (2021), through a large-scale longitudinal survey involving students from German universities, revealed that resilient individuals tend to have lower dropout intentions, achieve better grades, and report higher life satisfaction. Furthermore, Fullerton et al. (2021) found that resilience resources positively predict mental well-being and adjustment among Australian psychology undergraduate students.

Research on the factors and outcomes of university students’ academic resilience is particularly relevant in Lithuania, as studies in this area have not yet been developed to date. Investigating academic resilience in the Lithuanian context could complement studies conducted in other cultural settings and provide a foundation for exploring resilience among tertiary students in Lithuania.

The theoretical foundation of our study is built on several key aspects. The concept of academic resilience was grounded in the approach proposed by Martin and Marsh (2006). The resources related to academic resilience, encompassing both study-related and personal characteristics, as well as its positive outcomes, were analyzed within the framework of the Study Demands-Resources model (Mokgele and Rothmann, 2014; Bakker and Mostert, 2024; Lesener et al., 2020).

Our study aimed to explore the relationships between university students’ academic resilience, study resources, self-efficacy, engagement, and well-being. Two groups of resources were analyzed as predictors of student resilience: external resources, which included study characteristics such as autonomy, feedback, opportunities for professional development, support from peers and teaching staff, and internal personal resources, represented by student self-efficacy. Resilience outcomes were measured in terms of academic engagement and student well-being. Furthermore, resilience was examined as an intervening variable (mediator) in the relationships between study resources and student self-efficacy with resilience outcomes. The findings of the current research advance our understanding of university students’ academic resilience and contribute to the research of its resources and links to positive psychological outcomes.

## 2 Literature review

### 2.1 Academic resilience

APA Dictionary of Psychology defines resilience as “the process and outcome of successfully adapting to difficult or challenging life experiences, especially through mental, emotional, and behavioral flexibility and adjustment to external and internal demands”.^1^ A meta-analytic study on resilience (Hartmann et al., 2020) provides several definitions that generally conceptualize resilience as a person’s ability to support optimal functioning under challenging conditions and recover quickly from stressful situations. Luthar et al. (2000) define resilience as “a dynamic process encompassing positive adaptation within the context of significant adversity” (p. 543) and relate this personal characteristic to the ability to utilize contextual alongside personal resources to help a person withstand hardships, adapt to change, demonstrate flexibility, positive behavioral transformation in challenging situations, and positive adaptation in the context of adversity. According to Luthans (2002), resilience can be defined as “the developable positive psychological capacity to rebound, to “bounce back” from adversity, uncertainty, conflict, failure or even positive change, progress and increased responsibility” (p. 702). Masten (2014) emphasizes that resilience can be broadly described as „the capacity of a dynamic system to adapt successfully to disturbances that threaten system function, viability, or development” (p. 6). In the academic environment, resilience helps students overcome challenges, improve well-being, achieve academic success, and increase employment outcomes (Ang et al., 2021).

Baker et al. (2021) emphasize the complex nature and dynamism of resilience and its potential to be developed through experience. The dynamic nature of resilience as a personal strength implies that it is not infinite and can be depleted when used to cope with challenges or adversities. Therefore, according to the Conservation of Resources Theory (Hobfoll et al., 2018), resilience must be supported and supplemented by using external and strengthening internal personal resources. By emphasizing that individual resilience is a domain- or context-related feature (Fletcher and Sarkar, 2013) and is manifested within the interaction of the individual with a specific environment (Luthar et al., 2000), it becomes clear that the spectrum of factors that may impact resilience can include not only individual characteristics but also the conditions of the environment in which the activity is carried out (Masten, 2014).

After summarizing various definitions of resilience, several key characteristics emerge: it is a dynamic process involving positive adaptation to challenges; the ability to “bounce back” from adversity or failure; the ability to draw on both external and personal resources; and a developable psychological capacity supported by these resources. Since resilience is domain-specific and is manifested in the interaction of the individual with a specific environment (Luthar et al., 2000), this has led scholars to focus on resilience research in particular contexts and to explore resilience in different academic settings.

Students differ in how much they are impacted by study related pressures and failures, i.e., whether they can quickly recover from stressful situations, proactively react to poor results, and manage academic challenges. As Martin (2002) emphasizes, these differences are determined mainly by academic resilience, which is defined as “…students’ ability to deal effectively with academic setbacks, stress, and study pressure” (p. 35). Later, Martin and Marsh (2008) expanded the concept of resilience by distinguishing the phenomenon of buoyancy – resilience in everyday study situations. The authors define academic buoyancy as “students’ ability to successfully deal with academic setbacks and challenges that are typical of the ordinary course of school life (e.g., poor grades, competing deadlines, exam pressure, difficult schoolwork)” (p. 54). Studies have extensively analyzed the factors that enhance academic buoyancy, as well as their impact on academic achievements, student adjustment, and other study-related consequences (Datu and Yuen, 2018; Kritikou and Giovazolias, 2022).

In this study, we draw on the broader concept of academic resilience as the process of and capacity for successful adaptation in challenging or threatening academic circumstances (Martin, 2002). The impact of the same negative events, situations, failures, or even positive challenges on different individuals may be different. A negative assessment on a regular test may be a source of severe stress for one student, while another may view it as a challenge that motivates one to plan and differently organize the learning process, seek support, etc. Martin and Marsh (2006) also state that “…academic resilience is relevant to all students because at some point all students may experience some level of poor performance, adversity, challenge, or pressure” (p. 267).

Educational institutions at all levels strive to provide high-quality education, foster a supportive learning environment, promote student engagement and well-being, encourage retention, and minimize early dropout rates. However, the educational materials, conditions, and learning environments in primary and secondary schools, colleges, and universities can vary significantly. Therefore, it is important to study academic resilience in relation to the specific educational institution or even individual study programs. Research on the resilience of school and college students is increasingly gaining attention (Ye et al., 2024; Devi et al., 2019), but research on the academic resilience of university students is still lacking (Brewer et al., 2019). Entering university indicates a new period in students’ professional career. From the first days of study, they must adapt flexibly to new teaching and learning system, join a new community, and actively engage in the study process. High study results can be achieved if difficulties, challenges, or stressful situations that students may meet are effectively resolved and/or proactively managed. Although research on academic resilience among tertiary-level students is undoubtedly important, it is still underdeveloped.

We examined academic resilience among university students through two key areas of focus. The first focused on the resources that contribute to resilience, aiming to assess the study environment and personal characteristics that can enhance it. According to Fletcher and Sarkar (2013), it is essential to analyze a person’s immediate environment and “to develop the protective and promotive factors that individuals can proactively utilize to build resilience” (p. 18). Hartmann et al. (2020) also confirm that resilience-promoting factors are significant elements of the resilience process. It is, therefore, pertinent to explore the impact of the study environment and personal characteristics on university students’ academic resilience. Another focus of our study was associated with the potential role of academic resilience in predicting positive psychological outcomes – student engagement and well-being.

### 2.2 Resources of student academic resilience

Academic resilience can be examined from organizational and individual perspectives (Vercio et al., 2021). Resilience is expressed within a specific academic and social context, as enrolling in a university temporarily makes an individual both an active participant in the learning process and a member of the institution’s community. Therefore, from an organizational point of view, student academic resilience is a part of the overall resilience of the educational institution. According to Raetze et al. (2021), organizational resilience is shaped at individual, team, and organizational levels. Students, teaching and administrative staff, or the entire institution, may face various challenges or difficulties in their activities, which must be resolved or managed so as not to disrupt teaching, learning, other everyday work processes, or the functioning of the whole institution. Therefore, from a systemic point of view, examining and strengthening resilience at all the aforementioned levels is essential. In this study, we focused on individual-level student resilience, for which learning environments and conditions act as study contexts and may appear as protective forces.

We based our study on the Study Demands – Resources framework (Mokgele and Rothmann, 2014; Lesener et al., 2020; Bakker and Mostert, 2024), which is closely related to the widely known Job Demands – Resources (JD-R) theory (Bakker and Demerouti, 2017). Its principles are successfully applied in studies of the links between study demands and resources with academic engagement and burnout (Lesener et al., 2020; Cilliers et al., 2017; Zeijen et al., 2024). According to the Study Demands-Resources theory, the features of the study environment can be divided into two groups – demands and resources (Bakker and Mostert, 2024). Demands include various requirements related to academic activities – study load, the complexity of study material and tasks, the pace of learning, time constraints (Cilliers et al., 2017; Lesener et al., 2020), and even temporary difficulties, for example, related to COVID-19 lockdown circumstances (Martin et al., 2023).

Study resources include environment and personal characteristics that help students to meet learning requirements or overcome challenges and strengthen the student’s internal capabilities to achieve high learning outcomes, support high learning motivation and study-life balance (Bakker and Mostert, 2024). The wide range of study-related resources are most often examined as predictors of student engagement. Mokgele and Rothmann (2014) were among the first to apply the Study Demands–Resources model and confirmed that study resources (nature of the task, lecturer relations, and social support of peers) increased student engagement, which in turn positively predicted academic performance. Lesener et al. (2020) found that study resources (student support, teacher support, and developmental opportunities) strengthen student engagement and were negatively related to burnout. Van der Ross et al. (2022) explored the interplay of psychological conditions that influenced study engagement and conducted their survey in a sample of undergraduate students registered at a South African university. A strong association of study resources with student engagement was found. The range of study-related resources can be wider. For example, Martin et al. (2023) analyzed university students’ perceived adaptability and fluid reasoning as academic resources during the COVID-19 lockdown and revealed that both resources were positively linked with higher student engagement.

We included student autonomy, feedback, teacher and peer support, as well as opportunities for professional development as study-related resources contributing to academic resilience. Autonomy refers to opportunities given to students to independently organize and control the learning process and plan the time for individual learning. In a psychological sense, autonomy is the feeling that a person can predict and control their own learning and that “…one’s actions flow from one’s desires, and not merely from external demands” (Cullen and Oppenheimer, 2024, p. 1). Feedback refers to the process of receiving information about learning progress, results, and achievements. As Morris et al. (2021) state, feedback involves collecting and providing information about a student’s current performance with the aim to assist them in the learning process. Information about how well a student is doing in completing academic tasks can be received directly from the learning process, lecturers, or other students. When researching study resources, perhaps the greatest attention is paid to the social support factor. Support from lecturers reflects the possibility of getting advice, help, and encouragement from teachers in all study matters. Peer support describes practical help, informational or psychological support from other students if needed (Martin, 2002). Finally, opportunities for professional development refer to the academic program’s provisions for students to acquire high-quality knowledge, grow professionally, and enhance their skills and competencies (Mokgele and Rothmann, 2014; van Zyl et al., 2021; Van Zyl et al., 2021).

Self-efficacy was examined as a potential personal resource for students’ academic resilience. Self-efficacy refers to a person’s belief about their ability to mobilize motivation, cognitive, and other skills to act purposefully when necessary to fulfill situational demands (Bandura, 1982). Motivation, emotional states, and actions depend more on what people believe about themselves than what the objective truth is (Bandura, 2001). High efficacious people are more likely to view difficulties and obstacles as challenges to be overcome rather than obstacles to be avoided. Self-efficacy strengthens a person’s ability to self-motivate for activities, gather information, make reasonable decisions, and take proper actions, especially when faced with time pressure or other activity restrictions. According to research, self-efficacy is one of the most vital personal resources and predictors of academic resilience. Martin and Marsh (2006, 2008) found that self-efficacy significantly predicted academic resilience and buoyancy. Fullerton et al. (2021) analyzed academic efficacy as one of the personal resources of resilience alongside self-esteem, mental toughness, optimism, meaning in life, and adaptability. They revealed its positive links to undergraduate students’ adaptation outcomes (university adjustment and mental well-being). Cassidy (2015) also found that academic self-efficacy contributed to increased resilience in undergraduate students.

The inclusion of study and personal resources in the analysis of the assumptions of academic resilience provides an opportunity to comprehensively examine the factors of resilience and complement academic resilience studies that apply the Study Demands – Resources framework.

### 2.3 Academic resilience, student engagement, and well-being

#### 2.3.1 Academic resilience and student engagement

Engagement in studies, increasingly recognized as an essential influence factor on achievement and learning in higher education, is being widely theorized and researched. Schaufeli et al. demonstrated that school engagement could be considered a positive, fulfilling, study-related state of mind characterized by vigor, dedication, and absorption in learning (Schaufeli et al., 2002; Siu et al., 2014). Vigor refers to high levels of mental resilience while studying, an eagerness to invest effort, and a positive approach. A sense of significance, enthusiasm, pride, identification, meaning, and inspiration toward school characterize dedication.

Absorption is characterized by flow-like experiences, such as being so immersed in studies that time seems to pass quickly. Engagement represents an enduring and pervasive affective-cognitive state that focuses on specific and long-term activities (Schaufeli et al., 2002). A person may be engaged in one specific activity but not in others, so it is relevant to study the assumptions of student engagement by considering the activity’s nature, the environment, and other conditions.

According to the Study Demands – Resources theory, resources refer to enabling factors that promote student engagement. A series of studies have established that engagement in studies can be strengthened by internal proximal personal resources (e.g., self-efficacy) together with external distal resources, among which opportunities to develop professional competencies, feedback, autonomy, support from teachers and students can be mentioned (Cilliers et al., 2017; Lesener et al., 2020; van Zyl et al., 2024; Zeijen et al., 2024).

Studies also reveal that resilience, as students’ ability to withstand academic difficulties and support optimism, positive thinking, and problem-solving skills, significantly affects and predicts their engagement in the learning process (Ahmed et al., 2018; Sun and Liu, 2023; Versteeg et al., 2022). The behavior of academically resilient students is characterized by proactivity, when a person predicts difficulties that may arise, removes potential sources of their origin, foresees ways to cope with challenges or to manage them (Meneghel et al., 2019; Fullerton et al., 2021). Learning challenges or difficulties do not reduce the motivation and engagement of psychologically resilient students. On the contrary, overcoming difficulties becomes a source of positive emotions and satisfaction with the results achieved and can be an added motivator that encourages active involvement in studies.

#### 2.3.2 Academic resilience and student well-being

Student well-being was another potential consequence of university students’ academic resilience examined in our study. Studying at the university is a period of professional knowledge accumulation and competencies development, but personal growth and well-being are also important (Dodd et al., 2021; Hernández-Torrano et al., 2020). Huppert and So (2013) distinguish three main approaches in studying well-being: hedonic as subjective well-being (Diener, 2000), eudaimonic as psychological well-being (Ryff and Singer, 2008) and well-being as a combination of hedonic, eudaimonic and social well-being (Keyes, 2002). Huppert and So (2013) offered a conceptual framework in which flourishing or high well-being equates with positive mental health and can be identified by hedonic and eudaimonic aspects of well-being: competence, emotional stability, meaning, positive emotion, supportive relationships, self-esteem, and vitality (p. 837). To study university students’ well-being, we applied the World Health Organization’s proposed WHO – 5 Well-Being Index, which has been widely applied to assess subjective well-being in a variety of settings (Topp et al., 2015; Fung et al., 2022).

Studies indicate a positive relationship between academic resilience and psychological well-being and confirm that resilience may act as a protective factor that strengthens the students’ well-being, helping to avoid risk factors or reduce their negative impact (Chow et al., 2018; Idris et al., 2019; Malkoç and Yalçın, 2015). Low levels or even absence of academic resilience may negatively affect students’ mental health, increase psychological distress, and result in greater adjustment problems. According to Faye et al. (2018), it is expected that more resilient individuals will feel more psychological well-being. Some authors even claim that resilience can indicate mental health and well-being (Bittmann, 2021). Cassidy et al. (2023) conducted a large-scale study of the relationship between academic resilience and well-being in a sample of pharmacy education students. The results confirmed that resilience is related to and positively predicts student well-being. The authors also compared resilience indicators among student groups of different courses and found that resilience was highest in the first year and lowest in third-year student samples. The study used a cross-sectional strategy, which did not allow for drawing conclusions about the dynamics of resilience during studies as students move to a higher course. However, these results shed light on the perspective of longitudinal research on academic resilience.

### 2.4 The mediating role of academic resilience

In psychological research, the use of mediation models is applied to identify and explain the mechanism of the relationship between assumptions and effects via the inclusion of a mediating variable (Agler and De Boeck, 2017). Sarrionandia et al. (2018) conducted an intercultural study involving students from two universities in Japan and Spain and revealed that resilience mediated a negative relationship between emotional intelligence and stress experienced by students. Demirci et al.’s (2021) study of 14- to 18-year-old students found that resilience fully mediates the relationship between youth character strengths – wisdom, courage, optimism, and emotional balance – and psychological vulnerability. Meneghel et al. (2019) tested and empirically validated a theoretical model in which different stress-coping strategies were analyzed as antecedents of students’ academic resilience, academic satisfaction, and performance. Resilience acted as a mediator and explained the relationship between coping and academic satisfaction. Choo and Prihadi (2019) conducted a study on a sample of students from a Malaysian psychology undergraduate program and focused on the relationship between perfectionism and academic performance with academic resilience as a mediator. Hypotheses where academic resilience would mediate relationships between dimensions of perfectionism and academic performance were supported. Trigueros et al. (2020) analyzed the links between transformational teacher leadership, academic motivation, resilience, burnout, and academic performance. The sample consisted of more than three thousand university students from Spanish universities. The authors found that academic resilience positively mediated the relationship between teachers’ transformational leadership and academic performance and negatively with student burnout. Fiorilli et al. (2020) surveyed more than one thousand high school students and found that students with high emotional intelligence were more likely to exhibit resilience, which, in turn, reduced school burnout. Several studies can be mentioned that have identified the role of academic resilience as a mediator in the relationships between student self-efficacy and anxiety (Hayat et al., 2021) and personality traits and anxiety (Shi et al., 2015).

Regarding the role of academic resilience as a mediator when predicting engagement in studies and student well-being, it is essential to note that their number is quite limited, and only a few examples can be mentioned. In the sample of working students, Rahayu et al. (2024) confirmed that student resilience mediated the links of student self-efficacy and perceived social support with engagement in studies. Mahmoodimehr et al. (2023) investigated in high school students the association between health-oriented academic lifestyle and academic well-being via academic resilience as the mediator. The study revealed that academic resilience mediated the relationship between health-promoting and health-inhibiting behaviors with students’ well-being. Yu and Chae (2020) examined the relationships between medical students’ academic burnout and psychological well-being and determined the mediating role of resilience in this relationship. Zeng et al. (2016) surveyed students from primary, middle, and vocational schools and confirmed the mediating role of psychological resilience in the links of growth mindset with psychological well-being and school engagement. In our study, we sought to add to this field of study and tested the role of academic resilience as an intermediary variable (mediator) in the relationships between study resources and student self-efficacy with the two psychological outcomes – study engagement and well-being.

This study explores whether academic resilience mediates the links between study and personal resources with student engagement and well-being. This means that students who perceive their academic environment as providing autonomy and feedback, fostering the development of their skills, providing support from teachers and peers, and who have stronger efficacious beliefs may feel more resilient. In turn, resilient students may be more academically engaged and experience better well-being, expressed in a positive mood, activity, energy, and interest in things that fill daily life.

### 2.5 The current study

The main research phenomenon in our study was the academic resilience of university students. Its connections with study and personal resources, student engagement, and well-being were analyzed based on the Study Demands – Resources framework (Bakker and Mostert, 2024; Lesener et al., 2020). In this study, we focused on engagement and well-being as psychological outcomes of academic resilience, although its impact on both the student and the educational institution could extend far beyond this scope. In planning our study, we considered several aspects. Most academic resilience studies, which examine its assumptions and relationships with engagement in studies and well-being, have been conducted in primary and secondary school student samples. So far, a minimal number of studies have analyzed tertiary-level students’ academic resilience. It is also important to mention that there is not enough research analyzing academic resilience as a mediator in the relationship between study resources and student engagement and well-being.

**Figure 1:**
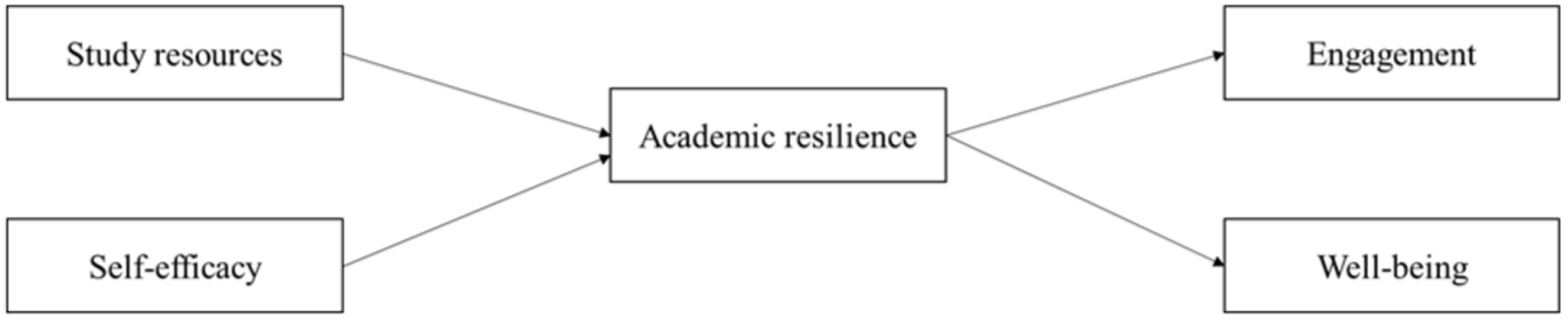
Research model.

In summary, this study aimed to explore the study and personal resources of university students’ academic resilience and its direct and mediating relationships with student engagement and well-being. The hypothesized research model is presented in Figure 1.

Research hypotheses:

*H1*: Study resources will be positively related to student academic resilience.

*H2*: Student self-efficacy will be positively related to academic resilience.

*H3*: Academic resilience will be positively related to student engagement.

*H4*: Academic resilience will be positively related to student well-being.

*H5*: Academic resilience will mediate the relationship between study resources and student engagement.

*H6*: Academic resilience will mediate the relationship between study resources and student well-being.

*H7*: Academic resilience will mediate the relationship between student self-efficacy and engagement.

*H8*: Academic resilience will mediate the relationship between student self-efficacy and well-being.

## 3 Materials and methods

### 3.1 Data collection procedure and participants

Data were collected using cross sectional study design, the convenience sample included 350 students from Lithuanian universities. Mirroring the gender distribution in Lithuanian tertiary-level institutions which is presented in the Overview of the 2024 general admission to Lithuanian higher education institutions by Association of Lithuanian Higher Education Institutions for Joint Admissions (LAMA BPO, 2024), most participants in our study were female (79.14%), the mean age was 23.8 years (SD =5.7). Students were in varying bachelor (69.1%), master (28,6%) and doctoral (2.3%) study programs. 62.0% of the respondents were employed – combined studies and work.

An online self-administered questionnaire was used to collect data; a survey was conducted in February – March 2024. Students were invited to take part in the survey by direct contact at lectures, via e-mails and social networks. Participants were also asked to share information about the survey with other students studying at the university.

In the cover letter, we presented the study’s purpose and gave instructions for completing the questionnaire. Participants were informed that the study was conducted following research ethics requirements, their participation was voluntary, responses were analyzed taken together for scientific purposes only, and that confidentiality of responses was guaranteed. Respondents could withdraw from the study at any time. Not a single respondent voluntarily withdrew from the study, and, finally, the responses of all 350 students were included in the data set. The research was approved by the Committee on Research Ethics in Psychology (No. 21 / (1.13 E) 250,000-KT-166, 2023-11-13).

### 3.2 Research instruments

The questionnaire included demographic questions on respondents’ age, gender, study program, a question about whether students are only studying or combining studies with work, and assessment scales for research variables.

*Academic Resilience* was assessed using six items scale (“I think I’m good at dealing with schoolwork pressures”) developed by Martin and Marsh (2006). Respondents evaluated each item on a five-point Likert scale ranging from 1 (“strongly disagree”) to 5 points (“strongly agree”). Academic resilience score was calculated as an average of answers to scale items, where higher scores refer to stronger academic resilience. The internal consistency of the scale was good (Cronbach’s alpha = 0.907). The exploratory factor analysis confirmed one-factor scale structure (Varimax rotation, KMO = 0.874, Bartlett test of sphericity Chi square = 1432.5, df = 15, *p* < 0.001) explaining 68.79% of variance with factor loadings ranging from 0.735 to 0.882.

*Study resources* were measured using a composite indicator consisting of five types of study-related resources (autonomy, feedback, student and teacher support, and opportunities for development) taken from the Job Demands–Resources Questionnaire (Bakker, 2014). Items were slightly changed and adapted for this study by changing the term “work” to “studies” in all scales. The term “colleagues” was changed to the term “students” in the student support scale and to “teachers” in the teachers’ support scale. *Autonomy* (“Can you participate in decision-making regarding your studies?”), *feedback* (“I receive sufficient information about the results of my studies”), *student support* (“Can you count on other students to support you, if difficulties arise in your studies?”), *teacher support* (“If necessary, can you ask teachers for help?”) and *opportunities for development* (“In my studies, I have the opportunity to develop my strong points”) were assessed using a three-item scale. Answers for autonomy, feedback, student support, and teacher support scales ranged from 1 point (never) to 5 (very often), and for opportunities for development, from 1 (strongly disagree) to 5 points (strongly agree).

Various characteristics of the study environment can be examined as individual variables and as aggregated latent constructs of study demands or resources, combining the study environment’s respective characteristics. For example, in the Lesener et al.’ (2020) research, challenging study demands, and time pressure served as indicators for the latent construct study demands, while student support, teacher support and opportunities for development were combined into a study resources indicator. In Mokgele and Rothmann (2014) research the study resources indicator included lecturer support, peer support, growth opportunities and information availability. In our study the five observed variables describing study resources (student autonomy, teacher support, student support, feedback and developmental opportunities) indicated the second-order latent construct “study resources.” The construct validity of the modeled study resources measure was evaluated by applying Principal component factoring with Varimax rotation when indicators of five study resource types were included as separate variables. Exploratory factor analysis showed the one-factor structure (KMO = 0.813, Bartlett test of sphericity Chi-square = 389.6, df = 10, *p* < 0.001), explaining 51.96% of the variance with factor loadings ranging from 0.665 to 0.768. Additionally, we conducted CFA for all 15 items while intercorrelating measurement errors of the same scale showed satisfactory fit: (χ^2^/df = 1.477, CFI = 0.985; GFI = 0.959; NFI = 0.957; TLI = 0.980; RMSEA = 0.037), 95% confidence interval CI (0.021–0.051). The internal consistency of the whole scale was good (Cronbach’s alpha = 0.931). To continue with further analysis, a mean score of five study resources variables was calculated as an individual indicator of study resources, with higher scores representing more study resources available for the student.

*Student Self-Efficacy* was assessed using a ten-item scale (“If I try hard enough, I can obtain the academic goals I desire”) developed by Rowbotham and Schmitz (2013). Respondents rated the items on a five-point Likert scale response format from 1 (strongly disagree) to 5 (strongly agree). An average score on the scale was calculated, with higher scores showing higher student self-efficacy. The internal consistency of the scale was good (Cronbach’s alpha = 0.848). The exploratory factor analysis confirmed one-factor scale (KMO = 0.871, Bartlett test of sphericity Chi square = 1,091, df = 45, *p* < 0.001) explaining 53.97% of variance with factor loadings ranging from 0.594 to 0.693.

*Student engagement* was measured with the Ultra-short Utrecht Work Engagement Scale (UWES-3) (Schaufeli et al., 2019) which is composed of three items representing three dimensions of the engagement: feeling energy (vigor), enthusiasm (dedication), and immersion (absorption). We chose the short study engagement scale because it is a reliable and valid research instrument, and our study focused on the overall student engagement and not on its sub-dimensions. The UWES-3 scale has been adapted in many countries, its psychometric properties have been confirmed in employee (Merino-Soto et al., 2022; Schaufeli et al., 2019) and student samples (Gusy et al., 2019; Versteeg et al., 2022; Ma and Wei, 2022). The original scale items were adapted by changing the term “work” to “studies”: “At my studies, I feel bursting with energy.” The response options were scaled from 1 (never) to 7 (always/every day). The internal consistency of scale was good (Cronbach’s alpha = 0.870). The exploratory factor analysis confirmed a one-factor scale’s structure (KMO = 0.729, Bartlett test of sphericity Chi-square = 539, df = 3, *p* < 0.001), explaining 79.7% of the variance with factor loadings ranging from 0.865 to 0.911.

*Well-being* was measured using the WHO-5 Well-being Index (Bech, 2004) which is among the most widely used questionnaires assessing subjective psychological well-being (Topp et al., 2015, p. 167). This is a generic five-item well-being scale developed for assessing well-being over a two-week period. It covers positive mood (feeling in good spirits, feeling relaxed), vitality (being active), and being interested in everyday things (Chow et al., 2018; Chan et al., 2022). The scale has been translated into more than 30 languages, validated across 35 countries (Sischka et al., 2020), tested in various populations with good psychometric properties, and has been widely applied across a wide range of study fields, including Lithuania (Topp et al., 2015; Jusienė et al., 2022). Respondents rated each item on a six-point Likert scale ranging from 1 (never) to 6 (always) regarding how they felt in the last two weeks (“I have felt calm and relaxed”). The WHO-5 Index was calculated as an average score of answers, the higher results represented the higher well-being. Exploratory factor analysis of subscales confirmed the one-factor structure (KMO = 0.850, Bartlett test of sphericity Chi-square = 971, df = 10, *p* < 0.001), explaining 68.87% of the variance with factor loadings ranging from 0.745 to 0.896. Scale’s Cronbach’s alpha in this study was 0.882.

Following Brislin’s (1986) recommendations, the questionnaire was administered in Lithuanian, translation of the items from English to Lithuanian was prepared by professional translators.

### 3.3 Statistical analyses

For this study, data analysis was carried out in three steps through IBM SPSS Version 21 and IBM SPSS AMOS Version 21 software. Primarily, we analyzed scales’ construct validity, reliability, and descriptive statistics (means, SD, Pearson correlations, Student’s t-test). Scales’ reliability was tested by calculating Cronbach’s alpha coefficients and construct validity – by applying exploratory and confirmatory factor analysis. Hierarchical regression analysis was used to examine predictors of academic resilience, study engagement, and well-being. A structural equation modeling (SEM) with 5.000 bootstrapping tests was conducted to test research hypotheses and to assess the effects of study resources and student self-efficacy on study engagement and well-being through academic resilience as a mediating variable. The structural equation model was evaluated through different goodness of fit (GOF) indexes: absolute fit indices (how well the prior model fits or reproduces the data); RMSEA (root-mean-square error of approximation), SRMR (standardized root mean square residual); GFI (goodness-of-fit index), and AGFI (adjusted goodness-of-fit index), incremental fit indices (deviation from the null model of representing factor); NFI (Normed fit index), TLI (Tacker-Lewis index), and CFI (Comparative fit index), and lastly the parsimonious fit indices (assessment of competing model); X2 (i.e., X2/degree of freedom). To reduce the potential harmful effects of common method bias (CMB), at the stage of data collection, the anonymity of the respondents was ensured. We also minimized common scale properties and used different response formats (five-, six-, or seven-point scale, measuring agreement with the items or frequency of the item content). Additionally, Harman’s single-factor (HSF) test to detect a common method bias in a dataset was conducted. The latter showed poor fit (χ^2^/df = 6.616, CFI = 0.468; GFI = 0.508; NFI = 0.430; TLI = 0.438; RMSEA = 0.127, 95% confidence interval CI (0.123, 0.130); factor loadings range from 0.297 to 0.696) so it is inferred that CMB is not a problem (Podsakoff et al., 2024).

## 4 Results

### 4.1 Descriptive statistics

Table 1 presents the means, standard deviations, and correlations between the study variables. All study variables had significant positive correlations with others, ranging from 0.406 to 0.536.

**Table 1:** Means, standard deviations, and correlations between variables (*N* = 350).

	*M*	*SD*	1	2	3	4
1. Study resources	3.73	0.61				
2. Self- efficacy	3.87	0.78	0.455^**^			
3. Academic resilience	4.56	1.25	0.406^**^	0.536^**^		
4. Student engagement	4.12	1.32	0.497^**^	0.525^**^	0.408^**^	
5. Well-being	3.29	0.98	0.369^**^	0.471^**^	0.508^**^	0.363^**^

**p < 0.01. M, mean; SD, standard deviation.

Correlations between variables reveal that associations among all analyzed characteristics are positive and significant. Correlations between academic resilience and other variables range from *r* = 0.406 (*p* < 0.01) for study resources to *r* = 0.536 (*p* < 0.01) regarding self-efficacy. Study resources correlate most strongly with student engagement, while self-efficacy and well-being are most strongly related with academic resilience.

Comparison of study variables by demographic characteristics revealed that males are more academically resilient (*M* = 4.98, *SD* = 1.18 vs. *M* = 4.45, *SD* = 1.25, t (111) = 3.278, *p* = 0.001) and their well-being is higher (*M* = 3.59, *SD* = 1.02 vs. *M* = 3.21, *SD* = 0.96, *t* (102) = 2.823, *p* = 0.006) comparing with females. It was found that age positively correlated with self-efficacy (*r* = 0.128, *p* = 0.017), academic resilience (*r* = 0.134, *p* = 0.012) and well-being (*r* = 0.140, *p* = 0.009). Comparing variables between undergraduate and graduate students’ groups did not show significant differences. Non-working students are slightly more engaged in their studies compared to students who combine studies and work: (*M* = 4.31, *SD* = 1.27 vs. *M* = 4.0, *SD* = 1.34, *t* (290) = 2.183, *p* = 0.03). When comparing other variables between groups by employment, no statistically significant differences were found.

### 4.2 Predictors of student academic resilience, engagement, and well-being

We tested six hierarchical regression models to examine predictors of academic resilience, student engagement, and well-being, with gender and age included as control variables in all models. For academic resilience as the dependent variable, control variables were included in Model 1, and study resources and self-efficacy as independent variables were included in Model 2. In Models 3 and 4, engagement as a dependent variable was regressed on control variables, study resources, self-efficacy, and resilience. In Models 5 and 6, well-being as a dependent variable was regressed on control variables, study resources, self-efficacy, and resilience. The results are presented in Table 2.

**Table 2:** Hierarchical regression models testing predictors of academic resilience, student engagement, and well–being.

Variables	Dependent variables					
	Academic resilience		Engagement		Well-being	
	Model 1	Model 2	Model 3	Model 4	Model 5	Model 6
Control variables	*β*	*β*	*β*	*β*	*β*	*β*
Gender	−0.172**	−0.135**	−0.003	−0.047	0.157**	0.082
Age	0.137*	0.086	0.133*	0.079	0.057	−0.015
Independent variables						
Study resources		0.223***		0.308***		0.139**
Self-efficacy		0.409***		0.319***		0.233***
Academic resilience				0.109*		0.315***
*R^2^*	0.047	0.343	0.018	0.376	0.027	0.334
Δ *R^2^*	0.047	0.296	0.018	0.358	0.027	0.306
*F*	(2, 344) 8.433**	(2, 342) 44.701***	(2, 344) 3.102*	(3, 341) 41.077***	(2, 344) 4.849**	(3, 341) 34.174***
Cohen’s *f^2^*	0.049	0.451	0.027	0.574	0.028	0.461

*p < 0.05; **p < 0.01; ***p < 0.001; *β*, standardized beta coefficient; VIF (Variance Inflation Factor) coefficients for the independent variables in every model did not exceed the statistical level of 2.0.

As shown by Model 1 and Model 2, after controlling for gender and age, the findings indicate that study resources and self-efficacy positively affected students’ academic resilience (*β* = 0.223, *p* < 0.001 and *β* = 0.409, *p* < 0.001, respectively) and explained 34.3% of the academic resilience variance, supporting H1 and H2. Data in Model 4 revealed that study resources, self-efficacy, and academic resilience positively affected student engagement (*β* = 0.308*, p < 0*.001, *β* = 0.319, *p* < 0.001 and *β* = 0.109, *p* < 0.05, respectively) and explained 37.6% of its variance. Academic resilience positively predicted student engagement supporting H3. Model 6 showed that study resources, self-efficacy, and academic resilience positively predicted student well-being (*β* = 0.139, *p* < 0.01, *β* = 0.233*, p* < 0.001 and *β* = 0.315, *p* < 0.001, respectively) and explained 33.4% of the well-being data variance. Academic resilience positively predicted student well-being supporting H4. The effect size of gender and age as control variables for dependent variables in all models is small (Cohen’s *f* ranged between 0.028 and 0.049). After adding independent predictors to the regression analysis, the effect size increased to large in predicting academic resilience (Cohen’s *f^2^* = 0.451), engagement (Cohen’s *f^2^ = 0*.574), and well-being (Cohen’s *f^2^ = 0*.461). Study resources, self-efficacy, and academic resilience accounted for 37.6% of the variance in student engagement and 33.4% in well-being.

The test of homogeneity of regression was conducted to estimate whether homogeneity of variance is not violated (Hayes and Preacher, 2014). The effects of study resilience on both outcomes (engagement and well-being) are invariant across both independent variables’ values: the *p-value* of the interaction of study resources and resilience in predicting engagement was 0.787, and in predicting well-being was 0.375; the *p-value* of the interaction of self-efficacy and academic resilience in predicting engagement was 0.720, and in predicting well-being was 0.619.

Regarding the fifth, sixth, seventh, and eighth hypotheses, we tested a model with direct and indirect relationships between study resources, self-efficacy, and student engagement and well-being. The mediating effects of academic resilience for the relationships between study resources and self-efficacy as independent variables and student engagement and well-being as dependent variables were examined by applying structural equation modeling (SEM) with 5.000 bootstrapping tests. The model’s fit to the data was tested as it is recommended in the scientific literature (Kline, 2005; Hu and Bentler, 1999), and showed an acceptable fit to the data:

- Chi-square χ^2^ = 1.332, degrees of freedom *df* = 1, *p* = 0.248; normed chi-square χ^2^*/df* = 1.332, that is less than 4.0 (it should be between 0 and 4 with lower values indicating a better fit);
- Comparative fit index *CFI* = 0.999; Goodness of fit index *GFI* = 0.998; *Adjusted GFI* = 0.977; the normal fit index *NFI* = 0.997; the Tucker-Lewis index *TLI* = 0.993 (all indices are above 0.95 and close to a value of 1.0 showing good support for the model structure);
- The root mean square error of approximation *RMSEA* = 0.031, 95% confidence interval CI (0.00–0.150); standardized root mean square residual *SRMR* = 0.010 (both should be between 0 and 0.08 with lower values indicating a better fit).

The AMOS mediation regression standardized weight estimates and their confidence intervals describing the total, direct, and indirect effects of study resources and self-efficacy on dependent variables are provided in Table 3.

**Table 3:** Direct, indirect, and total effects of study resources, self-efficacy, and academic resilience in predicting student engagement and well-being.

Independent variables	Mediator	Dependent variables	Direct effect	Indirect effect	Total effect
			*β*, 95% *CI*	*β*, 95% *CI*	*β*, 95% *CI*
Study resources	Academic resilience	Engagement	0.303 *** [0.205, 0.400]	0.022* [0.002, 0.056]	0.326*** [0.226, 0.426]
Study resources	Academic resilience	Well-being	0.128* [0.032, 0.225]	0.067*** [0.030, 0.116]	0.195*** [0.091, 0.300]
Self-efficacy	Academic resilience	Engagement	0.328*** [0.216, 0.434]	0.049* [0.001, 0.104]	0.377*** [0.281, 0.472]
Self-efficacy	Academic resilience	Well-being	0.237*** [0.143, 0.334]	0.146*** [0.094, 0.207]	0.383*** [0.290, 0.553]

*p < 0.05; **p < 0.01, ***p < 0.001, *β*, standardized beta coefficients, 95% CI, Confidence Intervals.

As Table 3 presents, the link between study resources and student engagement *via* academic resilience was significant (*β* = 0.022*, p* = 0.037, CI [0.002, 0.056]). While the total effect of study resources on engagement was significant (*β* = 0.326, *p* < 0.001), the direct effect was lower (*β* = 0.303, *p* < 0.001). Thus, academic resilience partly mediated the relationship between the study resources and engagement, supporting H5.

A significant mediating effect of academic resilience was revealed for the relationship between study resources and student well-being (*β* = 0.067 *p* < 0.001, CI [0.030, 0.116]). While the total effect of resources on well-being was significant (*β* = 0.195, *p* < 0.001), the direct effect after including the mediator decreased but remained significant (*β* = 0.128, *p* = 0.013). Thus, academic resilience partly mediated the relationship between the study resources and student well-being, supporting H6.

Consistent with H7, bootstrap mediation analysis showed that the relationship between self-efficacy and student engagement was mediated by academic resilience (*β* = 0.049, *p* = 0.047, CI [0.001, 0.104]). The total effect was significant (*β* = 0.377, *p* < 0.001), as well as the direct impact of self-efficacy on student engagement (*β* = 0.328, *p* < 0.001). Academic resilience partly mediated the relationship between student self-efficacy and engagement supporting H7.

**Figure 2:**
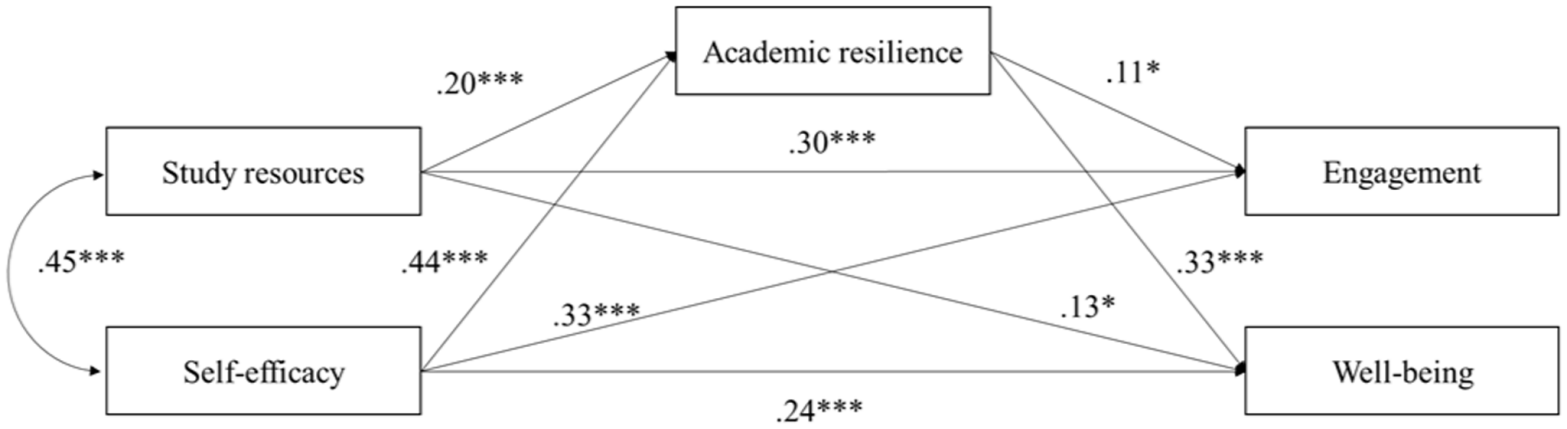
Empirical structural model presenting direct and indirect links between study resources and self-efficacy with student engagement and well-being *via* mediator academic resilience. *p < 0.05; **p < 0.01, ***p < 0.001, *β* (standardized beta coefficients) are included on the lines that show the established links between the study variables.

And finally, consistent with H8, bootstrap mediation analysis revealed that the relationship between student self-efficacy and well-being was mediated by academic resilience (*β* = 0.146, *p* < 0.001; CI: 0.094, 0.207). The total effect was significant (*β* = 0.383, *p* < 0.001), but the direct effect remained significant as well (*β* = 0.237, *p* < 0.001). That means, academic resilience partly mediated the relationship between self-efficacy and well-being supporting H8. The results are presented in Figure 2.

## 5 Discussion

The present study focuses on the academic resilience of university students, the antecedents of which and their links to positive outcomes are analyzed using the Study Demands – resources framework (Mokgele and Rothmann, 2014; Bakker and Mostert, 2024). The aim was to investigate study characteristics and self-efficacy as potential resources of students’ academic resilience and its links to student engagement and well-being. The external study resources variable was composed of autonomy, feedback, professional development opportunities, and peer and teaching staff support. Self-efficacy was analyzed as an internal personal resource. Positive resilience outcomes were represented by student engagement and well-being phenomena. More specifically, study resources and student self-efficacy were analyzed as predictors of academic resilience (hypotheses H1 and H2). Furthermore, academic resilience was examined as a predictor of student engagement (hypothesis H3) and well-being (hypothesis H4) and as an intervening variable (mediator) in the relationships of study resources and student self-efficacy with engagement (hypotheses H5 and H7) and well-being (hypotheses H6 and H8).

We found that study resources and self-efficacy positively affected students’ resilience. Both predictors explained 34.3% of the resilience variance. However, the impact of self-efficacy on resilience (*β* = 0.409, *p* < 0.001) was stronger compared with external study resources (*β* = 0.223, *p* < 0.001) (see Table 2, Model 2). Hypotheses H1 and H2 were confirmed: more resilient were students who had more autonomy (opportunities to organize and control the learning process), received appropriate feedback (information about students’ learning progress and achievements), were supported by other students and teachers with advice, encouragement, or emotional help, had more opportunities to improve in the professional field, and were more efficacious. These results confirm the studies that reveal the importance of different contextual characteristics in promoting academic resilience: high-quality relations and social support (Frisby et al., 2020), environmental protective factors, including technology, physical learning environment, and academic support (Cobb et al., 2024), positive school climate and a supportive school environment (Hunsu et al., 2023). The identified links between students’ self-efficacy and resilience also replicate the results obtained in studies by other authors. For example, Martin and Marsh (2006, 2008) found self-efficacy to predict academic resilience and buoyancy significantly. Fullerton et al. (2021) revealed that self-efficacy as one of the personal resources of resilience was positively linked to undergraduate students’ university adjustment and mental well-being. Cassidy (2015) also found that academic self-efficacy contributed to increased resilience in undergraduate students. Hughes et al. (2021) state that it is important to include educational strategies to enhance student nurses’ internal protective factors of resilience, self-efficacy, optimism, and emotional intelligence in nursing students’ education.

Checking the links between academic resilience and student engagement, we found that resilience, study resources, and self-efficacy positively predicted student engagement (see Table 2, Model 4) and explained 37.6% of the engagement variance. Hypothesis H3 was confirmed: students who rate their academic resilience higher are more engaged in a positive, fulfilling, study-related state of mind characterized by vigor, dedication, and absorption in learning. However, the effect of academic resilience on engagement is smaller (*β* = 0.109, *p* < 0.05) than study resources (*β* = 0.308, *p* < 0.001) and self-efficacy (*β* = 0.319, *p* < 0.001). Regarding the positive associations of resilience with engagement, the results of our study support those received in studies of Ahmed et al. (2018), Sun and Liu (2023), Versteeg et al. (2022), and other authors.

It was also found that academic resilience, study resources, and self-efficacy were significant predictors of student well-being (see Table 2, Model 6). All three independent variables explained 33.4% of the well-being variance. Hypothesis H4 was confirmed: students who rated their resilience higher also reported higher psychological well-being. In this case, resilience is a more substantial prognostic factor than study resources and self-efficacy as its impact on well-being is stronger (*β* = 0.315, *p* < 0.001) compared to study resources (*β* = 0.139, *p* < 0.01) and self-efficacy (*β* = 0.233, *p* < 0.001).

The positive relationship of academic resilience with student well-being found in our study partially confirms the results of other studies. Fullerton et al. (2021), in a sample of 306 undergraduate students, examined resilience resources and their interaction with coping responses to produce positive adaptation outcomes (mental well-being, university adjustment, and somatic health symptoms). Resilience was included in the composite resilience resources indicator along with self-esteem, academic self-efficacy, adaptability, and life orientation. It was established that resilience resources relate to mental well-being via social support as a mediator. According to studies conducted by Faye et al. (2018), Bittmann (2021), Cassidy et al. (2023), individuals who are more resilient feel more psychological well-being.

When evaluating the mediating relationships between the analyzed phenomena, four hypotheses were put forward about the role of academic resilience as a mediator in the relationships of study resources and self-efficacy with engagement and well-being. The received results confirm that academic resilience partially mediated the relationships between study resources and engagement (H5), resources and well-being (H6), as well as relationships between self-efficacy and both dependent variables – engagement (H7) and well-being (H8). Study resources and self-efficacy were indirectly related to higher student engagement and well-being due to the higher resilience of students.

These results confirm and complement the few studies by other authors that examine the role of resilience as a mediator in predicting student engagement (Rahayu et al., 2024), academic well-being (Mahmoodimehr et al., 2023), psychological well-being (Yu and Chae, 2020), psychological well-being and engagement (Zeng et al., 2016). However, it is important to note that the largest number of studies, whose authors delve into the importance of academic resilience as a mediator in the interrelationships of various phenomena, examine resilience in the context of stress and its coping issues (Sarrionandia et al., 2018; Hayat et al., 2021).

In summary, the direct impact of study environment-related factors and self-efficacy on academic resilience is positive; both can strengthen resilience and be described as significant resources for academic resilience. Resilience enhances student engagement and well-being directly and thus acts as a mediating variable of the relationship between resilience resources and these positive psychological outcomes. Students who perceive their academic environment as providing autonomy and feedback, fostering the development of their skills, providing support from teachers and peers, and who have stronger efficacious beliefs may feel more resilient. In turn, resilient students may be more academically engaged and feel higher well-being, expressed as a positive mood, activity, energy, and interest in things that fill daily life.

## 6 Limitations and directions for future studies

The present study provides data on the under-researched topic of academic resilience among university students, exploring its connections with study and personal resources, student engagement, and well-being. While the results contribute to the existing body of research, addressing the study’s limitations is essential, highlighting opportunities for further research in this field.

One limitation of this study is the unequal gender distribution in our sample. The evidence for our findings was drawn from a homogeneous group of Lithuanian university students, with female students significantly overrepresented. This imbalance is not unusual, as gender-related differences in survey participation and nonresponse are well-documented, particularly in mail and web-based surveys. Research shows that women are more likely than men to take part in surveys (Slauson-Blevins and Johnson, 2016; Keusch, 2015). Therefore, further studies are needed to investigate how our findings can be generalized to students with more diverse demographic characteristics. Participants in this study were selected based on accessibility; however, using a convenience sampling strategy limits the representativeness of the general population of Lithuanian university students. To address this limitation, future studies should consider employing stratified sampling methods to ensure representation of the entire student population in higher education institutions. Additionally, to reduce homogeneity in terms of age, gender, or other demographic factors, researchers could use personalized invitations, send reminders emphasizing the value of participants’ contributions, and enhance interest in the study by shortening questionnaires or offering small incentives.

Our study relied on students’ self-reports as the only source of information. The survey items focused on individuals’ subjective perceptions of their ability to maintain emotional balance in challenging situations and use external and personal resources to overcome setbacks. To address this limitation, studies could incorporate methods that capture students’ daily experiences, such as the diary method, to provide more nuanced and context-rich data.

The data were collected using a cross-sectional design. Therefore, future research should consider applying longitudinal designs to assess the stability of resilience and other psychological variables examined here (self-efficacy, engagement, and well-being) over time. Learners progress through their studies, therefore replicated research could help explore the dynamics of resilience and its links with resources, and outcomes. As educational material and lecturers change, the psychological climate, relationships with counterparts, and other resources provided by the university environment may also change. The learning experiences gained during studies may strengthen personal and professional competencies, providing opportunities to develop new skills for overcoming challenges. These factors may contribute to enhancing students’ academic resilience and promote constructive behaviors in demanding situations. A longitudinal research design could offer deeper insights into how these factors influence academic resilience. It is also important to note that resilience and the analyzed outcomes may be mutually reinforcing, as engagement and well-being can, in turn, enhance resilience in the long term.

Future research should focus on a broader range of personal and study environment variables and their relationships with student academic resilience. For example, student motivation or professional calling warrants more in-depth exploration. Expanding the diversity of academic resilience resources by incorporating additional personal characteristics (e.g., personality traits or proactivity) and study environment factors (e.g., teacher leadership style or communication with students) is important. The Study Demands-Resources model includes resources and addresses study demands such as study workload or cognitive demands. The complex relationship between study demands and resources with academic resilience has only been analyzed in high school student samples. Therefore, applying this comprehensive model to study academic resilience in tertiary-level students is highly relevant. Regular student surveys grounded in the full Study Demands-Resources model could help higher education institutions continuously build a valuable database, analyze the dynamics and drivers of academic resilience, and implement empirically based interventions.

The identified links between resilience, academic engagement, and student well-being contribute to the limited body of research exploring the positive outcomes of academic resilience. For instance, this study found that academic resilience positively relates to student engagement; however, resilience is a weaker predictor of engagement than study resources and self-efficacy. This finding opens up opportunities for further research into the prognostic factors of student engagement and well-being and a more detailed examination of the connections between academic resilience, engagement, and well-being.

## 7 Implications

Our study complements research in the field of academic resilience in several aspects. We base our study on the Study Demands – Resources theory (Bakker and Mostert, 2024; Lesener et al., 2020), which allows researchers to analyze various external and personal factors as resources of academic resilience. So far, this model is more widely applied in the workplace, studying the assumptions of employee engagement and burnout, while the number of studies applying this model in the study environment is limited. According to this model, in studies, as in other activities, the resources that a student can use help to implement the demands and achieve performance results and enhance such positive psychological outcomes as motivation, engagement in learning activities, or commitment to studies. Resources can also positively affect the individual’s internal strengths, which help them to adapt to the learning environment, overcome the challenges of study, learn from experience, and improve. Resilience is one of the learners’ internal strengths, the support and sustainable development of which begins with answering the question – what study and personal resources are important for the student’s academic resilience in the concrete learning environment?

This study provides insights into the interplay between academic resilience, its resources, academic engagement, and well-being and complements earlier research in several ways. First, the results expand existing research on the study and personal resources of university students’ academic resilience. Second, the mechanism through which resources are related to student engagement and well-being is revealed, suggesting that academic resilience is an intermediate factor (mediator) in this relationship. Third, in this study, student academic resilience was measured using a scale developed by Martin and Marsh (2006), which has been used more often in studies of the resilience of secondary school students. The obtained results confirm that this measure can be applied when studying the resilience of university students, and the collected data opens opportunities for further validation of this instrument in Lithuania.

This study was conducted on a sample of Lithuanian university students, but the cultural context and conditions in which students study in higher education institutions in other countries differ. Therefore, when studying the prerequisites of tertiary-level students’ academic resilience, it is relevant to apply the Study Demands – Resources framework more widely and to develop comparative studies of the prerequisites and consequences of university-level students’ academic resilience in other countries.

Our results have practical implications for increasing students’ academic resilience and improving their engagement and well-being through enhancing academic resilience. The following aspects that relate to resilience-building interventions can be distinguished.

The first is an individual approach related to strengthening students’ awareness of their personal resources and protective role in supporting mental health and overcoming study difficulties. This includes developing stress coping, problem-solving, effective communication, and other competencies and skills, applying adaptation programs for newly enrolled students, providing emotional support, and mentoring. University counselors and other mental health professionals should give students preventive interventions on resilience programs and treating psychological problems. University counseling centers might provide psycho-educational programs for first-year students and all students to increase their psychological well-being. For these mental health initiatives to make a meaningful impact, universities should strengthen policies aimed at involving students themselves in mental health promotion; students must feel encouraged to use counseling services or self-help interventions. As a person’s well-being is inseparable from other areas of an individual’s life, academic resilience can be important for wider student life domains – work or family life.

Resilience-strengthening interventions should not only focus on developing students’ competencies and skills. Students become a constituent part of the university as an organization for a certain period. Thus, another approach focuses on organizational-level interventions. Students learn in a specific environment; they are influenced by many study environments and other factors related to the study process, which form the learning context. It is essential to take care of student autonomy, feedback, support from teachers and peers, and development opportunities related to academic programs, as those organizational factors can have a double opposite effect on students’ academic resilience – to strengthen it or to become sources of constant stress or tension that reduce students’ reserves of resilience.

It is emphasized that investing in students’ academic resilience, engagement, and mental well-being is not only a moral imperative for universities but also essential for the organization’s long-term achievements. Therefore, to effectively strengthen students’ academic resilience, it is important to monitor the level of resilience and their opinions about the learning environment at least once during the academic year. Interventions must be planned and implemented complexly when measures for students are combined with measures that create favorable conditions for studies and learning. By investing in the study environment and personal resources of students’ academic resilience, universities can create a more inclusive, supportive, and successful learning environment, ensuring their engagement in studies and well-being.

## 8 Conclusion

Academic resilience is a psychological characteristic that explains the links between study and personal resources with engagement in studies and well-being. Understanding antecedents of academic resilience and psychological processes linking study and personal resources with students’ engagement and well–being offers the opportunity to develop interventions that might strengthen university students’ academic resilience and might help to increase engagement in studies and well-being.

Young people at university-level studies meet new study demands, which are related to a significant risk of psychological distress and mental health problems. Examining the resources of university students’ academic resilience – both study and personal – and its relationship with student engagement and well-being in a complex way is essential as it offers the opportunity to develop proper interventions. However, academic resilience research has mainly focused on high school students. Our study, guided by the Study Demands–Resources framework, aimed to fill a gap in academic resilience research by focusing on university students.

We aimed to reveal personal and environmental resources that are closely related to university students’ academic resilience, which functions as a factor affecting student engagement and well-being. Our results showed that study resources and self-efficacy positively predicted student academic resilience; study resources, self-efficacy, and academic resilience positively predicted student engagement and well-being; academic resilience mediated the relationships of study and personal resources with student engagement and well-being. Students who value the resources provided by the university, have confidence in themselves, believe in their abilities to perform study tasks effectively, are more engaged in their studies, and highly evaluate their well-being. This underscores the pivotal role of academic resilience in the university context.

Our findings prove the need for a more comprehensive consideration of the learning environment and conditions at the university-level educational institution. This is crucial for protecting and enhancing students’ academic resilience and strengthening their decision and motivation to stay at university, continue their studies, and generally feel well in the educational environment. The obtained results also open perspectives for further research on academic resilience at the university level studies.

The findings offer valuable insights into the study and personal resources contributing to university students’ academic resilience. They highlight the pivotal role of resilience in mediating the interplay between resources, engagement, and well-being. These insights have practical implications for fostering student resilience, enhancing engagement, and improving well-being by strengthening both study-related and personal resources.

## Data Availability

The raw data supporting the conclusions of this article will be made available by the authors, without undue reservation.

## Footnotes

^1^ https://dictionary.apa.org/resilience
